# Validation and Meaningful Change Thresholds for an Objective Cough Frequency Measurement in Chronic Cough

**DOI:** 10.1007/s00408-022-00587-2

**Published:** 2022-11-08

**Authors:** Jonathan Schelfhout, Allison Martin Nguyen, Surinder S. Birring, Elizabeth D. Bacci, Margaret Vernon, David R. Muccino, Carmen La Rosa, Jaclyn A. Smith

**Affiliations:** 1 Merck & Co., Inc., Rahway, NJ USA; 2 Centre for Human and Applied Physiological Sciences, School of Basic and Medical Biosciences, Faculty of Life Sciences and Medicine, King’s College London, London, UK; 3 Evidera, Seattle, WA USA; 4 Evidera, Bethesda, MD USA; 5 Division of Infection, Immunity and Respiratory Medicine, 2nd Floor Education and Research Centre, University of Manchester and Manchester University NHS Foundation Trust, Southmoor Rd, Wythenshawe, Manchester, M23 9LT UK

**Keywords:** Chronic cough, Clinically meaningful change, Cough monitoring, Cough severity, Objective cough frequency, Patient-reported outcomes

## Abstract

**Purpose:**

Objective cough frequency is used to assess efficacy of chronic cough (CC) treatments. The objective of this study was to explore the relationship between objective cough frequency and cough-specific patient-reported outcomes (PROs) and estimate a clinically meaningful change threshold (MCT) for objective cough frequency.

**Methods:**

Data collected in a phase 2b study in participants with refractory or unexplained CC were used to investigate the relationship between 24-h cough frequency (measured using an ambulatory cough monitor) and cough-specific PROs (i.e., cough severity visual analog scale, cough severity diary, Leicester Cough Questionnaire). Convergent validity was assessed using Spearman *ρ*. An MCT for 24-h cough frequency was estimated using the patient global impression of change (PGIC) scale as an anchor.

**Results:**

Correlations between 24-h cough frequency and cough-specific PROs at baseline, Week 4, and Week 12 were significant (*P* < 0.0001) but low to moderate in strength (*ρ* = 0.30–0.58). Participants categorized as *very much improved/much improved* (i.e., PGIC of 1 or 2) or *minimally improved* (i.e., PGIC of 3) had mean 24-h cough frequency reductions of 55% and 30%, respectively. Receiver operating characteristic curve analysis suggested that a 24-h cough frequency reduction of 38% optimizes sensitivity and specificity for predicting a PGIC score of 1–3.

**Conclusion:**

Objective 24-h cough frequency is significantly associated with cough-specific PROs, but cough frequency and PROs most likely capture distinct aspects of CC. A ≥ 30% reduction in 24-h cough frequency is a reasonable MCT to define treatment response in CC clinical trials.

**Supplementary Information:**

The online version contains supplementary material available at 10.1007/s00408-022-00587-2.

## Introduction

Chronic cough (CC) is defined as a cough lasting more than 8 weeks, though some patients with CC experience a cough that occurs daily or almost daily for several years [1–5]. Patients with CC cough frequently, with some patients coughing several dozen times per hour while awake [6–8]. Prolonged and frequent coughing can exert substantial burden on patients, inducing negative effects on physical, social, and psychological well-being [3, 4, 9], and cause high healthcare utilization due to repetitive medical visits and treatment trials [3, 10–12]. Although treatment of associated medical conditions (e.g., asthma, allergic rhinitis, gastroesophageal reflux disease) can resolve cough, a subset of patients with CC have a cough that persists despite optimal diagnosis and management of associated conditions according to published guidelines [i.e., refractory CC (RCC)] or a cough that persists despite a lack of identifiable, treatable conditions associated with cough after extensive investigation [i.e., unexplained CC (UCC)] [1, 2, 13]. The lack of approved treatments with indications for RCC or UCC reflects a major unmet need.

Monitoring and treating CC is a relatively new field—within the past 2 decades, both objective and subjective tools for assessing cough have been developed [14]. Objective cough monitoring using ambulatory devices to assess reductions in cough after treatment with antitussives has been used in several recent clinical trials in RCC or UCC [6, 7, 15–19], as well as in studies of other respiratory conditions, including acute cough, asthma, and chronic obstructive pulmonary disease (COPD) [20–22]. Although cough monitoring is important for evaluating objective efficacy of novel antitussives, this technique is not currently widely available outside of clinical trial settings [14]. As the goal of managing CC in a clinical setting is to improve patients’ symptoms, cough-specific patient-reported outcomes (PROs), such as the cough severity visual analog scale (VAS), cough severity diary (CSD), and Leicester Cough Questionnaire (LCQ), have been developed and validated to monitor various aspects of cough [14, 23]. Because PROs are designed to capture patient-reported aspects of cough (e.g., severity, frequency, intensity, disruption), these subjective tools provide complementary insights into objective cough monitoring when evaluating efficacy of antitussive therapies.

Although both objective cough monitoring and cough-specific PROs have been used in clinical practice and clinical trials, few studies have investigated the relationship between cough frequency and cough-specific PROs [23–25]. Clinically meaningful change thresholds (MCTs) for some cough-specific PROs have been established [23, 26, 27], but a within-patient MCT for objective cough frequency has not been estimated. Therefore, we conducted this analysis to evaluate the relationship between objective cough frequency and cough-specific PROs and to estimate an MCT for 24-h cough frequency in patients with RCC or UCC.

## Methods

### Study Design

For this analysis, we used data from a phase 2b study of the P2X3-receptor antagonist gefapixant (ClinicalTrials.gov identifier: NCT02612610) [6]. Eligible participants had RCC or UCC (per American College of Chest Physicians and British Thoracic Society guidelines) lasting ≥ 1 year and a cough severity VAS ≥ 40 mm. Candidates were excluded if they were current or recent (within 6 months of enrollment) smokers or had substantial chest abnormalities contributing to cough as determined by a chest x-ray within the past 5 years. No eligibility criteria were based on cough frequency. Participants from all treatment groups (i.e., gefapixant and placebo groups) were pooled for this analysis to assess the measurement properties of 24-h cough frequency.

### Outcome Measures

Objective cough frequency was measured as previously described [6]. An ambulatory acoustic recording device (VitaloJAK™; Vitalograph Ltd, Buckingham, England) was used to collect sound recordings over 24-h periods, and cough frequencies were assessed by trained analysts after removal of silence and noncough sounds using custom written software. Twenty-four-h cough frequency was calculated as the total number of coughs over the 24-h period divided by 24. There are previous reports with detailed information on the development and validation of the device and software and reliability of the trained analysts [28, 29].

Additional outcomes collected in the phase 2b study included the cough severity VAS, CSD, LCQ, and patient global impression of change (PGIC) scale. The cough severity VAS records patients’ self-assessment of cough severity on a 100-mm linear scale ranging from *no cough* (0 mm) to *worst cough* (100 mm) [27]. The CSD is a 7-item questionnaire that assesses the frequency, intensity, and disruptiveness of a patient’s cough on an 11-point scale ranging from 0 to 10, with higher scores indicating greater cough severity; the total CSD score is calculated as the mean of individual item scores [23]. The LCQ assesses cough-specific health-related quality of life (HRQOL) using 19 individual items across physical, social, and psychological domains; each item is measured on a 7-point Likert scale, and the total score is calculated as the sum of individual domain scores (range, 3–21; lower scores reflect worse cough-specific HRQOL) [9]. The PGIC measures overall patient-reported improvement on a 7-point scale ranging from 1 (*very much improved*) to 7 (*very much worse*).

Cough frequency, cough severity VAS, and LCQ scores were measured at baseline and Weeks 4, 8, and 12 [6]. The CSD score was calculated weekly as the average of the 7 preceding daily CSD scores and was collected from baseline to Week 12. The PGIC was assessed at Weeks 4, 8, and 12. Participants were categorized into 5 distinct groups based on their PGIC ratings at Weeks 4 and 12: PGIC of 1 or 2 (*very much improved* or *much improved*), PGIC of 3 (*minimally improved*), PGIC of 4 (*no change*), PGIC of 5 (*minimally worse*), or PGIC of 6 or 7 (*much worse* or *very much worse*).

### Statistical Analysis

All statistical tests in this analysis used a significance level of *P* < 0.05.

#### Convergent Validity

Convergent validity (or association of related measures) was investigated by comparing 24-h cough frequency with cough severity VAS, CSD, and LCQ at baseline and Weeks 4 and 12. Nonparametric Spearman rank correlation (Spearman *ρ*) was used for cross-sectional correlations because of the non-normal distribution of cough frequency. Low-to-moderate correlations were expected, as the measures capture similar but conceptually different information.

#### Known-Groups Validity

To assess known-groups validity of 24-h cough frequency, participants were stratified into severity groups based on the sample distribution at baseline using 2 metrics of severity. The first was based on tertiles of CSD total scores; the second was based on LCQ total score categories of ≤ 8, > 8 and ≤ 13, and > 13. Analysis of variance was used to assess for significant differences in 24-h objective cough frequency between the severity groups, with post hoc category comparisons via the Scheffé test.

#### Score Interpretation

Anchor- and distribution-based approaches were used to evaluate within-patient MCTs for 24-h cough frequency. Anchor-based estimates of MCTs compared changes in 24-h cough frequency across categories of the 7-point PGIC (1, *very much improved*; 2, *much improved*; 3, *minimally improved*; 4, *no change*; 5, *minimally worse*; 6, *much worse*; 7, *very much worse*). An MCT was defined as the mean 24-h cough frequency reduction in participants who reported themselves as *minimally improved* on the PGIC (PGIC of 3). A second anchor-based approach to estimate an MCT was conducted using a receiver operating characteristic (ROC) curve analysis to determine the threshold value for change in 24-h cough frequency from baseline to Week 4, defined as the point on the ROC curve closest to 100% sensitivity and specificity for predicting participants scoring 1–3 on the PGIC (i.e., the ROC point with the shortest difference from the upper left quadrant [0, 1]). Youden index was used to determine the change in 24-h cough frequency that optimized sensitivity and specificity for predicting global improvements on the PGIC.

Distribution-based approaches included one-half of SD and SE of measurement (SEM) at baseline. The former method is calculated as one-half the SD observed at baseline and has been suggested as a good approximation of the minimally important difference [30]. The SEM is calculated by multiplying the baseline SD by the square root of (1 − intraclass correlation coefficient [ICC]), where ICC is test–retest reliability from baseline to Week 4.

Estimates for the within-patient MCT for 24-h cough frequency were made by triangulating results of the anchor- and distribution-based approaches. The agreement between the MCT for 24-h cough frequency estimated in this analysis and the published MCT for the CSD was assessed using the kappa statistic (*κ*) for agreement between categorical data. This analysis was conducted to compare responder rates defined using a subjective measure (CSD) versus an objective measure (cough frequency). The strength of agreement, reflected by *κ*, was interpreted using previously published benchmarks [31].

## Results

### Study Population

Baseline characteristics of participants enrolled in this study were previously published [6, 32]. Mean (SD) baseline scores were 29.5 (39.4) coughs/h for 24-h cough frequency, 57.5 (22.3) mm for cough severity VAS, 4.3 (1.9) points for CSD total score, and 11.7 (3.0) points for LCQ total score.

### Convergent Validity

**Fig. 1:**
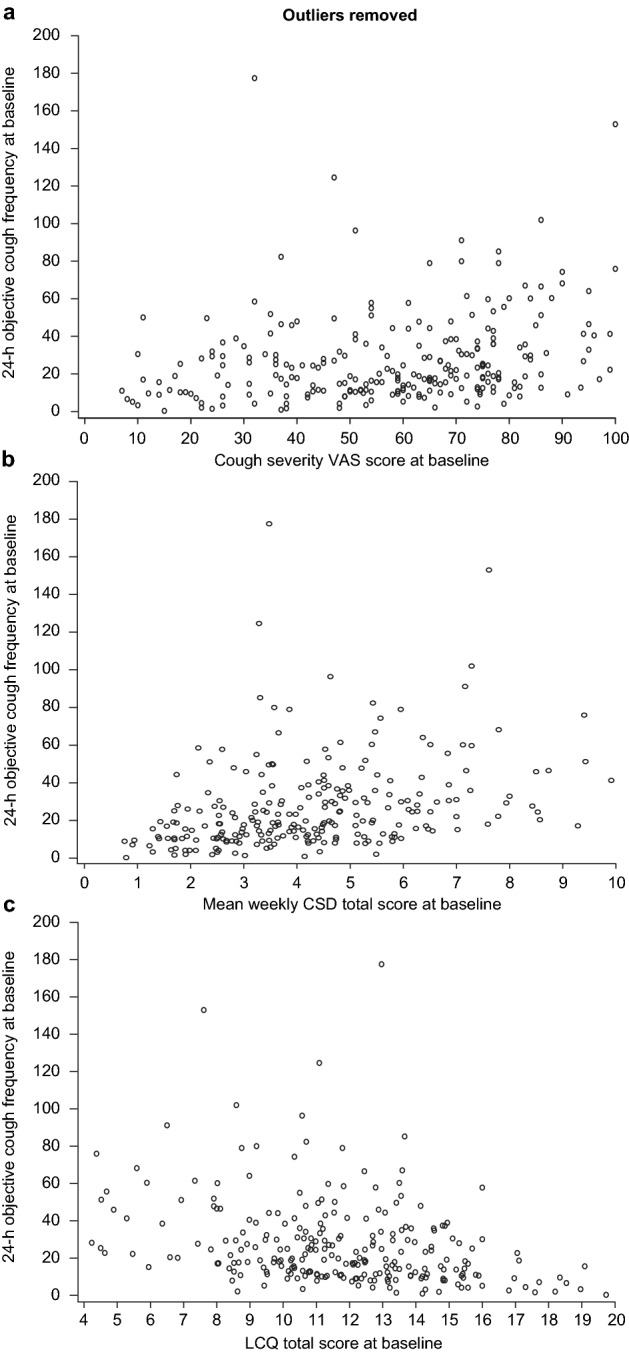
Scatter plots (with outliers removed) of 24-h cough frequency versus **a** cough severity VAS, **b** mean weekly CSD total scores, and **c** LCQ total scores. One outlier data point has been removed from each plot to aid visualization. CSD, cough severity diary; LCQ, Leicester Cough Questionnaire; VAS, visual analog scale

Correlations were assessed between 24-h cough frequency and cough-specific PRO total and domain scores at baseline and Weeks 4 and 12 using Spearman *ρ* (Table 1; Fig. 1). Correlations were low to moderate at each cross-sectional time point.

**Table 1:** Convergent validity of 24-h cough frequency with cough-specific PROs

Cough-specific PRO	Spearman *ρ* with 24-h cough frequency		
	Baseline	Week 4	Week 12
Cough severity VAS	0.30	0.49	0.54
CSD (total)	0.43	0.57	0.48
CSD (frequency)	0.45	0.58	0.49
CSD (intensity)	0.40	0.55	0.46
CSD (disruption)	0.31	0.46	0.43
LCQ (total)	− 0.37	− 0.58	− 0.56
LCQ (physical)	− 0.42	− 0.54	− 0.54
LCQ (psychological)	− 0.31	− 0.55	− 0.55
LCQ (social)	− 0.31	− 0.55	− 0.50

All correlation coefficients reflect significant correlations (*P* < 0.0001)

CSD, cough severity diary; LCQ, Leicester Cough Questionnaire; PRO, patient-reported outcome; VAS, visual analog scale

### Known-Groups Validity

In support of known-groups validity, baseline mean 24-h cough frequencies increased with increases in CSD total scores (*F* = 7.70; *P* = 0.0006; Table 2). Additionally, mean 24-h objective cough frequencies were lowest in participants with the highest LCQ total scores (indicating better cough-specific HRQOL) and increased with decreasing LCQ total scores (*F* = 15.56; *P* < 0.001).

**Table 2:** Known-groups validity at baseline by CSD and LCQ total score groups

	CSD total score							
	Tertile group 1		Tertile group 2		Tertile group 3		Overall *F* value	*P* value
	*n*	Mean (SE)	*n*	Mean (SE)	*n*	Mean (SE)		
24-h objective cough frequency	75	20.7 (4.5)	85	25.6 (4.3)	77	44.2 (4.5)	7.70	0.0006

	LCQ total score							
	Score ≤ 8		Score > 8 to ≤ 13		Score > 13		Overall *F* value	*P* value
	*n*	Mean (SE)	*n*	Mean (SE)	*n*	Mean (SE)		
24-h objective cough frequency	25	66.8 (7.5)	141	28.9 (3.1)	85	19.5 (4.1)	15.56	< 0.0001

CSD, cough severity diary; LCQ, Leicester Cough Questionnaire

### Score Interpretation

Anchor-based estimates of within-patient MCTs were conducted using PGIC category as an anchor. There was a trend toward greater reductions in 24-h cough frequency for groups reflecting greater improvements according to the PGIC (Table 3). Participants who reported themselves as *minimally improved* (PGIC of 3) at Weeks 4 and 12 had a reduction in 24-h cough frequency of approximately 30%, reflecting a much greater change than the approximately 2–6% reductions observed in the *no change* PGIC group (PGIC of 4) over the same time periods. Participants with greatest improvements according to the PGIC (PGIC of 1 or 2) had the highest reductions in 24-h cough frequency (Week 4, 58%; Week 12, 55%). Similar results were observed when examining trends in awake cough frequency reductions across PGIC categories (Online Resource 1).

**Table 3:** Change in 24-h cough frequency from baseline to Weeks 4 and 12 by PGIC category

PGIC category	Change in 24-h cough frequency, Week 4			Change in 24-h cough frequency, Week 12		
	*n*	Mean (SD) change, coughs/h	Percentage change (%)	*n*	Mean (SD) change, coughs/h	Percentage change (%)
PGIC 1 and 2	83	− 16.5 (17.6)	− 57.5	107	− 14.0 (22.1)	− 55.4
PGIC 3	76	− 10.4 (20.8)	− 29.7	53	− 9.7 (23.4)	− 27.3
PGIC 4	59	− 4.2 (14.6)	− 6.6	48	− 4.4 (24.9)	− 2.2
PGIC 5^a^	5	2.8 (12.4)	6.5	12	3.1 (9.7)	24.7
PGIC 6 and 7	4	5.1 (5.3)	24.8	–	–	–

PGIC, patient global impression of change

^a^PGIC ≥ 5 for change in 24-h cough frequency at Week 12

**Fig. 2:**
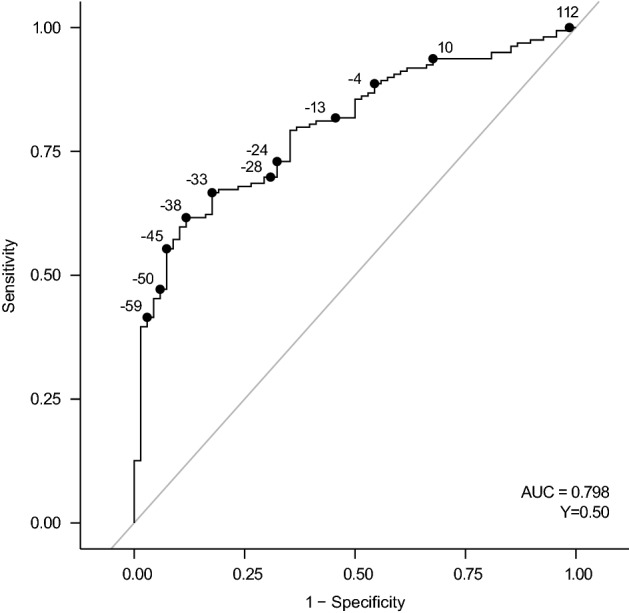
Receiver operating characteristic curve for predicting PGIC score of 1–3 on the basis of percentage change in 24-h cough frequency at Week 4. AUC, area under the curve; PGIC, patient global impression of change; Y, Youden index

An ROC analysis was also conducted at Week 4 using a PGIC score of 1–3 as an anchor (Fig. 2). Sensitivity and specificity in predicting a PGIC score of 1–3, as measured by the Youden index, was optimized at a 38% reduction in 24-h cough frequency (Table 4).

**Table 4:** Receiver operating characteristic curve analysis for 24-h cough frequency thresholds predictive of PGIC score of 1–3 at Week 4

24-h cough frequency reduction (%)	Sensitivity	Specificity	PPV	NPV	Youden index
≤ 10	0.85	0.50	0.80	0.59	0.35
≤ 20	0.77	0.65	0.84	0.54	0.41
≤ 30	0.68	0.74	0.86	0.50	0.41
≤ 38^a^	0.62	0.88	0.92	0.50	0.50
≤ 40	0.60	0.88	0.92	0.48	0.48
≤ 50	0.47	0.94	0.95	0.43	0.41
≤ 60	0.40	0.97	0.97	0.41	0.37
≤ 70	0.31	0.99	0.98	0.38	0.29
≤ 80	0.16	0.99	0.96	0.33	0.14
≤ 90	0.07	1.00	1.00	0.31	0.07

NPV, negative predictive value; PGIC, patient global impression of change; PPV, positive predictive value

^a^Represents the cough frequency reduction at when Youden index was maximized

Mean (SD) 24-h cough frequency at baseline was 29.5 (39.4) coughs/h. Estimates for the distribution-based MCT were 19.7 (one-half of SD) and 15.9 (SEM) coughs/h.

A ≥ 30% reduction in 24-h cough frequency, based on triangulating threshold estimates from anchor-based analyses, was identified as a potential MCT. The proportion of participants who met this responder definition for 24-h cough frequency was compared with the proportion of participants who were responders according to a previously estimated MCT for the CSD (i.e., ≥ 1.3-point reduction in total CSD score [23]). Among 125 participants who were responders by the 24-h cough frequency responder definition at Week 4, 83 (66%) were also responders on the CSD. Among 87 participants who were nonresponders by the 24-h cough frequency responder definition at Week 4, 57 (65%) were also nonresponders on the CSD. The kappa statistic for agreement between measures was consistent, with fair agreement between the 24-h cough frequency and CSD responder definitions (*κ* = 0.31; *P* < 0.0001). Similar agreement was observed for the ≥ 30% reduction in 24-h cough frequency responder definition with both the cough severity VAS (≥ 30-mm reduction) and LCQ (≥ 1.3-point increase) responder definitions at Week 4. Among participants with cough severity VAS data, 57% (72/127) of cough frequency responders were cough severity VAS responders and 81% (75/93) of cough frequency nonresponders were cough severity VAS nonresponders. Among participants with LCQ data, 81% (102/126) of cough frequency responders were LCQ responders and 57% (58/102) of cough frequency nonresponders were LCQ nonresponders.

## Discussion

To our knowledge, this is the first analysis formally estimating an MCT for 24-h cough frequency in patients with RCC or UCC. This analysis demonstrates that 24-h cough frequency is moderately but significantly correlated with cough-specific PROs and estimates a ≥ 30% reduction as an MCT for 24-h cough frequency.

Objective cough monitoring and cough-specific PROs capture distinct yet complementary aspects of cough, with PROs capturing subjective evaluations of cough, which can vary greatly among patients (even among those with similar cough frequencies depending on the perceived effect of cough on their daily lives). Consistent with this consideration, the correlation coefficients between 24-h cough frequency and cough-specific PROs observed in the current study were low to moderate in strength when analyzed at a single time point and were similar to previously reported correlations between cough frequency and cough-specific PROs [24, 33, 34]. Additionally, relationships between cough frequency and cough severity VAS and cough frequency and LCQ in the current analysis strengthened over time, suggesting that improvements in cough frequency may be closely correlated to improvements in other aspects of cough (e.g., cough-specific HRQOL) than comparisons at single time points. Ultimately, this study reinforces that objective and subjective measures of cough are complementary and, when taken together, describe the multifaceted impact of cough on patients.

An anchor-based approach using a participant-reported minimal change on the PGIC (PGIC of 3) suggested an approximate MCT of a 30% reduction, whereas participants reporting themselves as *very much improved* or *much improved* (PGIC of 1 or 2) had reductions in 24-h cough frequency of approximately 50%. The ROC curve–based analysis (which categorized a response as a PGIC score of 1–3) suggested a change threshold for 24-h cough frequency between these 2 estimates (i.e., an approximate 40% reduction). Meanwhile, distribution-based estimates produced a more robust MCT (i.e., 16–20 coughs/h, or an approximate 50–70% reduction given the baseline mean 24-h cough frequency of 30 coughs/h) due to the large variability in cough frequency observed in the patient population. Notably, an MCT expressed as a percentage rather than absolute reduction in cough frequency was considered more meaningful given that percentage reductions are a standard measure for reporting in CC clinical trials, inherently adjust for variation in baseline cough frequency, and are more interpretable from a clinical perspective. An MCT based on absolute change would have poor generalizability to those with higher or lower baseline cough frequencies.

Overall, these data suggest a minimum MCT of 30% as an appropriate target when assessing cough frequency reduction after antitussive therapy, though use of more stringent thresholds may also be reasonable. In primary findings from the clinical trial used as the source for the current analysis, 80% of participants treated with gefapixant 50 mg were responders by the ≥ 30% reduction threshold (vs. 44% of participants who received placebo) [6]. Moreover, 51% of participants treated with gefapixant 50 mg had a ≥ 50% reduction in awake cough frequency (vs. 25% in the placebo group). These data suggest reduction thresholds of 30% and 50% are achievable outcomes in clinical trials assessing novel treatments of RCC or UCC. Further studies investigating whether the 30% cough frequency reduction threshold can discriminate between effective and noneffective interventions for RCC or UCC are warranted.

There are limitations to the current study. First, all enrolled participants were from the USA or UK and had moderate to severe (i.e., baseline cough severity VAS ≥ 40 mm) and long-lasting CC. Therefore, confirming these findings in a broader geographic patient population and among patients with less severe CC or more recent-onset CC is warranted. Second, as this trial enrolled participants with RCC or UCC, the MCT determined in this analysis may not directly apply to patients with other causes of CC (e.g., COPD). Finally, the timeline for assessing meaningful change was extended through only 12 weeks of treatment, and analyses using longer treatment periods may be warranted to examine the stability and consistency of these results.

In conclusion, this analysis provides insights into the relationship between objective cough frequency and subjective cough-specific PROs for monitoring CC and provides MCT estimates for use in CC clinical trials or clinical practice.

## Supplementary Information

Below is the link to the electronic supplementary material.

Supplementary file 1 (PDF 63 KB)
