# Supplementary material for: Validation and Meaningful Change Thresholds for an Objective Cough Frequency Measurement in Chronic Cough
Source: Lung. 2022 Nov 8;200(6):717–24. doi: 10.1007/s00408-022-00587-2 (PMC9675653; doi:10.1007/s00408-022-00587-2)
Supplement: Supplementary file 1 — (PDF 63 KB) [file 408_2022_587_MOESM1_ESM.pdf]

ONLINE RESOURCE 1

Supplemental Table. Change in Awake Cough Frequency From Baseline to Weeks 4 and 12 by PGIC Category

	Change in awake cough frequency, Week 4			Change in awake cough frequency, Week 12		
**PGIC category**	**n**	**Mean (SD) change, coughs/h**	**Percentage change**	**n**	**Mean (SD) change, coughs/h**	**Percentage change**
PGIC 1 and 2	83	-22.8 (23.5)	-57.8%	107	-18.4 (36.3)	-54.2%
PGIC 3	76	-13.5 (31.1)	-27.9%	53	-12.8 (35.1)	-27.2%
PGIC 4	59	-6.9 (25.0)	-8.1%	48	-6.4 (34.2)	-2.5%
PGIC 5[a]	5	-1.6 (15.9)	-1.9%	12	5.3 (11.9)	23.8%
PGIC 6 and 7	4	6.7 (5.1)	26.4%	—	—	—

PGIC, patient global impression of change. [a]PGIC ≥5 for change in awake cough frequency at Week 12.

From: Schelfhout et al. Validation and meaningful change thresholds for an objective cough frequency measurement in chronic cough. *Lung.* Corresponding author: Jaclyn A. Smith (Jacky.Smith@manchester.ac.uk)
